# A Complete Genome Sequence of *Podosphaera xanthii* Isolate YZU573, the Causal Agent of Powdery Mildew Isolated from Cucumber in China

**DOI:** 10.3390/pathogens12040561

**Published:** 2023-04-06

**Authors:** Ziyi Wang, Yujiao Du, Suhao Li, Xuewen Xu, Xuehao Chen

**Affiliations:** 1 School of Horticulture and Landscape Architecture, Yangzhou University, Yangzhou 225009, China; wrince73@163.com (Z.W.); dd18752595989@163.com (Y.D.); hli808863@gmail.com (S.L.); 2 Joint International Research Laboratory of Agriculture and Agri-Product Safety, The Ministry of Education of China, Yangzhou University, Yangzhou 225009, China

**Keywords:** *Podosphaera xanthii*, YZU573, cucumber, genome assembly

## Abstract

*Podosphaera xanthii* is a well-known obligate biotrophic pathogen that causes powdery mildew (PM) disease on cucurbitaceous plants and is one of the most important limiting factors for cucumber production worldwide. To better understand the avirulence effector proteins in this species that are known to be involved in host-pathogen interaction, the draft genome assembly of *P. xanthii* isolate YZU573 from cucumber leaves with symptoms of PM was obtained with a hybrid approach, combining nanopore long-read and llumina paired-end sequencing. The final *P. xanthii* YZU573 genome assembly of 152.7 Mb consists of 58 contigs, with an N50 value of 0.75 Mb and 6491 predicted protein-coding genes. The effector analysis using the whole-genome sequence information revealed a total of 87 putative effector candidates, and 65 of them had their analogs, whereas the remaining 22 were novel ones. The new *P. xanthii* genome provides valuable resources to better understand plant-microbe interaction in cucumber PM disease.

## 1. Introduction

Cucurbit powdery mildew (PM), which is mainly caused by the obligate biotrophic pathogen *Podosphaera xanthii* (synonym *Podosphaera fusca*), is a serious disease affecting field and greenhouse cucurbits worldwide, including cucumbers, melons, watermelons, squash, gourds, and pumpkins [1,2]. The first sign of PM is white, powdery-like patches or spots on leaf and petiole surfaces. These spots will enlarge quickly until the entire tissue is covered. When environmental conditions become unfavorable, cleistothecium is formed. Since the report of *P. xanthii* in California in 1925, more than 28 physiological races have been identified according to their reactions to differential melon accessions [3,4]. The predominant race of PM populations depends on cultivars, growing season, and geographical area [5]. Races 1, 2, and 5 are the most prevalent in southern European regions [6,7]. In China, races 1 and 2F are the major races, with race 2F as the prevailing race causing melon PM in Beijing [8,9].

The most economical way to control PM is to breed new resistant cultivars through a breeding program with the introgression of major resistance genes (*R* genes) from resistant lines, but the PM resistance in cucumber is complicated: different organs showed varied levels of PM resistance including cotyledons, hypocotyls, stem, true leaves, seedlings, and mature plants [10,11]. Instead, genome-editing of susceptibility genes, which encode products exploited by pathogens through effector proteins during infection and colonization, has been proposed as an alternative to *R* genes in breeding cucumbers resistant to PM [12,13]. Increasing evidence suggests a crucial role for the effector proteins secreted by fungi in controlling pathogenesis into host cells [14]. Recent advances in high-throughput genome sequencing technologies and bioinformatics-based predictions have facilitated the identification of effector proteins in pathogenic organisms for subsequent experimental validation [15]. The previous two sequenced genomes of *P. xanthii* have been reported using Illumina and Pacbio reads [16,17]. However, the physiological races of the two sequenced *P. xanthii* isolates were not identified. In addition, little is known about the effector repertoire of *P. xanthii*. Here, we report the draft genome sequence of the cucumber PM disease pathogen, *P. xanthii* race 2F, assembled with a hybrid de novo genome assembly approach.

## 2. Materials and Methods

### 2.1. Pathogen Infection and Phenotype Evaluation

A single PM conidium was collected with a sterilized needle from PM susceptible D8 (a semi-dwarf American-type cucumber inbred line) leaves in a greenhouse in Yangzhou city of China. The PM conidia were inoculated on the surface of D8 cotyledons grown in growth camber with an interior light intensity of 250 μmol m^−2^ s^−1^, a humidity of 80% ± 5%, and temperature of 28 °C/24 °C (12 h/12 h) light/dark. The *P. xanthii* isolate was named YZU573. The physiological race of YZU573 was determined in a greenhouse by the reactions of 13 melon (*Cucumis melo* L.) accessions, which included Iran H, Top Mark, Vedrantais, PMR 45, PMR 5, WMR29, Edisto47, PI 414723, MR-1, PI124111, PI124112, PMR6, and Nantais Oblong [4]. Melon plants at the ten-leaf stage were inoculated with freshly prepared YZU573 spore suspension (1 × 10^6^ spores per mL in 0.01% Tween-20) collected from D8 seedlings. Disease severity was evaluated according to a previously described disease index (DI)-based method at approximately 60 days after planting when D8 had clear PM symptoms. The experiments were repeated over three years with 20–25 plants for one accession per year, according to a completely randomized experimental design.

### 2.2. Library Preparation and Sequencing

We extracted high molecular weight-genomic DNA from the epiphytic parts of the PM mycelium from D8 cotyledons with the methods provided by Feehan et al. [18] and Li et al. [19]. After the quantity and quality check procedure by NanoDrop™ One (Thermo Scientific, Wilmington, DE, USA), 4 μg DNA was sequenced to obtain both long and short reads using two sequencing platforms, nanopore and Illumina. In the case of nanopore, a library was constructed using the Oxford Nanopore ligation sequencing kit (SQK-LSK109, Oxford Nanopore Technologies, Oxford, UK) protocol and sequenced through an R9 flow cell (FLO-PRO002, Oxford Nanopore Technologies, Oxford, UK) on nanopore PromethION48 (Oxford nanopore technologies, Oxford, UK) for 72 h. In the case of Illumina, a stand Illumina sequencing library was constructed using the llumina DNA Library Prep kit that was later sequenced with Illumina Novaseq6000 (Illumina, San Diego, CA, USA) to produce 200 bp pair reads. We amplified the internal transcribed spacer (ITS) sequence from YZU573 DNA with the primer pair PxT (5′-TTTGGCGGGCCGGGCTCGACC-3′) and ITS4 (5′-TCCTCCGCTTATTGATATGC-3′) [20]. The PCR amplification had an initial denaturation step at 95 °C for 2 min, followed by 30 identical cycles at 95 °C for 30 s, 55 °C for 30 s, and 72 °C for 30 s, and a final hold step at 72 °C for 5 min before a 4 °C hold. The 25 μL PCR reaction mixtures constituted of 2 μL of DNA template (100 ng/μL), 1.5 μL of PxT primer (10 μM), 1.5 μL of ITS4 primer (10 μM), 12.5 μL of 2 × TaqPCR mix (Vazyme Biotech Co., Ltd., Nanjing, China), and 7.5 μL of ddH_2_O. The experiment was repeated three times to confirm the results. 

### 2.3. Genome Assembly and Annotation

The nanopore sequencing reads were base-called with quality filtering (--min_length 1000 --min_mean_q 7) using filtlong v0.2.1 (https://github.com/rrwick/Filtlong, accessed on 18 October 2022). All Illumina reads were filtered with fastp v0.20.0 [21] and then used to correct errors in the nanopore reads with LoRDEC [22] and Canu v2.2 [23]. We mapped all of the sequencing reads to the cucumber genome assembly (http://cucurbitgenomics.org/v2/organism/19, accessed on 19 October 2022) to filter cucumber sequences. The remaining nanopore long reads were then used for genome assembly with NextDenovo v. 2.0 (https://github.com/Nextomics/NextDenovo, accessed on 19 October 2022). After assembly, the single base accuracy is further polished with minimap2 v2.1 [24], NextPolish v1.4.0 [25], and pilon v1.23 [26]. The completeness of the genome assembly was evaluated with BUSCO (benchmarking universal single-copy orthologs) v9 [27]. The genome size and heterozygosity were estimated from Illumina paired-end sequences by Kmer profiling using GenomeScope 2.0 [28]. 

Protein-coding genes were predicted using the Augustus v3.3.3 [29] pipeline with the default parameters. The ribosomal RNAs (rRNAs) were predicted using RNAmmer v1.2 [30], the transfer RNAs (tRNAs) were predicted using tRNAscan-SEAbyss v1.23 [31], and other small RNAs (sRNAs) were identified using the Rfam_scan.pl v1.0.4 against Rfam database v 11.0 [32]. RepeatModeler v2.0.1 [33] and RepeatMasker v4.1.0 [34] were used to quantify the extent of repetitive sequences and transposable elements. Functional annotation of the protein-coding genes was carried out by performing blast (v.2.2.3.1) (E-value  ≤  10^−5^) searches against entries in the following seven major public databases: the non-redundant protein (NR) database, Swiss-Prot protein sequence database, Kyoto Encyclopedia of Genes and Genomes (KEGG) database, clusters of orthologous groups for eukaryotic complete genomes (KOG) database, Clusters of Orthologous Groups (COG) database, Gene Ontology (GO) database, and the protein families (Pfam) database.

### 2.4. Identification of Effector Proteins

To identify putative effector proteins, all proteins were checked for the presence of signal peptides using SignalP 4.1 [35] and the absence of transmembrane domain using TMHMM v2.0 [36]. The resulting sequences were extracted and submitted to EffectorP-fungi v3.0 [15] for the prediction of final candidate effector proteins.

## 3. Results and Discussion

### 3.1. Pathogen Isolation and Physiological Race of YZU573

**Figure 1:**
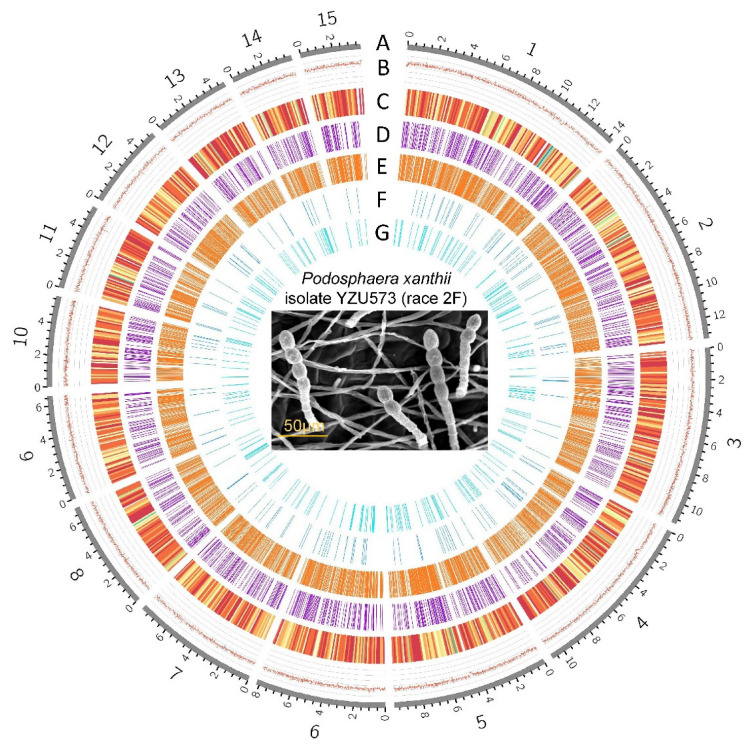
The genomic features of *Podosphaera xanthii* YZU573 (race 2F). The genome size of isolate YZU573 is 152,748,770 bp. A, Contigs (the top 15 in length are shown). B, GC contents. C, Distribution of the protein-coding genes. D, Distribution of 1778 pathogen-host interaction genes. E, Distribution of the 1724 secreted proteins. F, Distribution of the 191 genes encoding carbohydrate enzymes. G, Distribution of the 499 noncoding RNAs.

Differences among the 13 melon accessions upon YZU573 inoculation are listed in Table 1. A total of 3 of the 13 accessions, including Edisto47, PI414723, and WMR29, were highly resistant to YZU573 inoculation. However, PMR45 was highly susceptible to YZU573 inoculation, with average DI ranging from 93.5 to 112.3. The summary reaction pattern of the differentials in this test was identical to that for *P. xanthii* race 2F [37,38]. Sanger sequencing and BLASTn analysis of the amplified sequence from conidia of isolate YZU573 with the primer pair PxT/ITS4 showed a 100% match with the ITS sequence of *P. xanthii* on melon (KP980563) from Southeast China [20]. Microscopic observations further confirmed the isolate as *P. xanthii* by observation of conidial and appressorial shapes (Figure 1) [39]. Our results support the previous conclusion that *P. xanthii* race 2F is the predominant physiological race in most areas of China [4].

### 3.2. Genome Assembly 

In the case of nanopore, we obtained a total of 5,581,682 reads (mean read length of 6049 bp). In the case of Illumina, we obtained a total of 2,437,846,921 reads. The final reference genome assembly size of YZU573 is 152,748,770 bp, which contained 58 contigs, with an N50 value of 749,368 bp, and the longest contig was 14,306,468 bp. The average GC content was 43.27%, while the repetitive sequences comprise 72.39% of the genome sequence (Figure 1). The BUSCO genome integrity score was 99.2%, while only 0.4% was partially presented, providing support for a high level of gene completeness (Table 2). BLAST analysis revealed that the contig46 contains nuclear ribosomal DNA (nrDNA) sequences with high similarities to the 5.8S (OQ552886), 18S (MK225523), and 28S (MK225554) nrDNA sequences determined by Sanger sequencing of *P. xanthii* in NCBI GenBank nucleotide database. These basic metrics indicate the high quality of this de novo assembly. The assembled genome of this study was less in size than the published *P. xanthii* genome (209,067,775 bp) from cucumber [16] but was higher than the *P. xanthii* isolate 2086 genome (142,114,041 bp) from zucchini [17]. However, the degree of continuity was higher in YZU573 than in the assembly by Kim et al. [16] with N50 of 581,650 bp or the assembly by Polonio et al. [17] with N50 of 163,173 bp.

**Figure 2:**
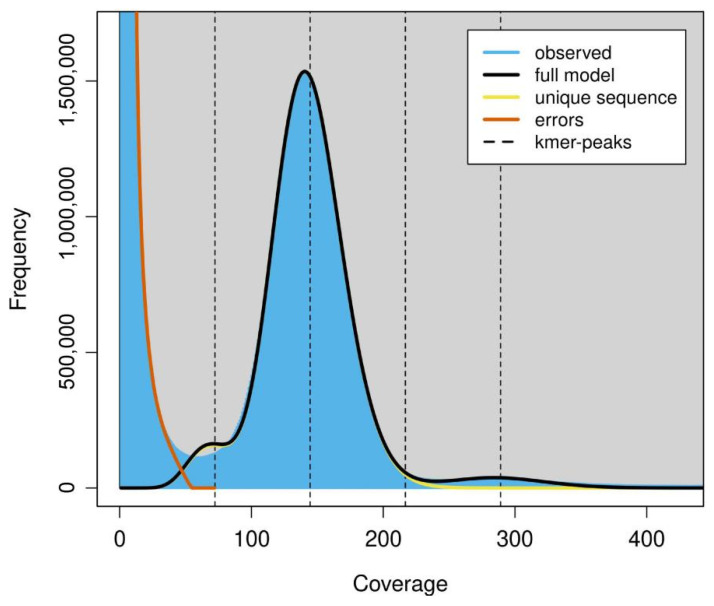
The k-mer frequency of Illumina short reads. Plot was generated by GenomeScope using k = 75. The fit of the GenomeScope model (black line) and the k-mer frequencies observed (blue area). K-mer coverages (x-axis) were plotted against the value of coverage multiplying frequency (y-axis).

The size of the YZU573 genome estimated by GenomeScope ranged from 150,156,306 bp to 150,362,206 bp with a 67.5% unique sequence (Figure 2). The estimation was comparable with that of our draft genome (152,748,770 bp), further suggesting that this could be a “quality reference” genome.

### 3.3. Annotation of YZU573 Genome

**Figure 3:**
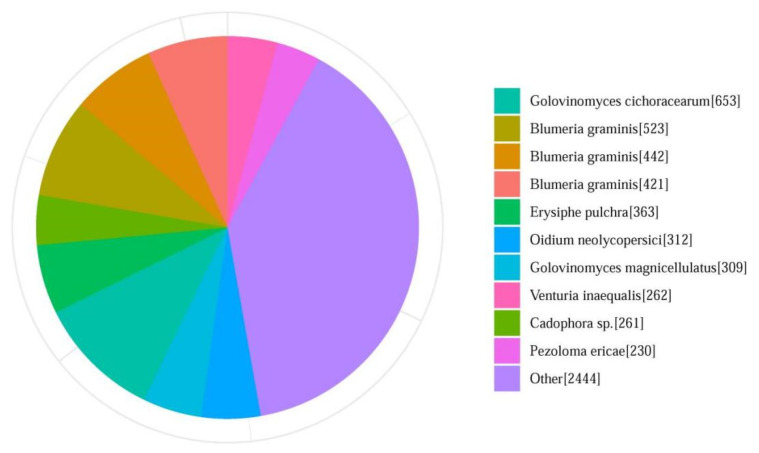
Summary of homologous species distributions in the Non-Redundant (Nr) Protein Database. Genes that had BLAST annotations within the Nr database with an E-value cut-off of ≤10^−5^ were used for species distribution.

In total, 72.39% of the assembled YZU573 genome was annotated as transposable elements (TEs), including long interspersed nuclear elements (LINEs; 40.41%), long terminal repeat (21.22%), DNA transposons (10.75%), and short interspersed nuclear elements (0.01%). The results showed that LINEs are the most abundant TEs in the *P. xanthii* genome. After masking the repeat elements, a total of 6491 protein-coding genes and 499 noncoding RNAs, including 423 tRNAs, 12 rRNAs, and 64 sRNAs, were predicted. These genomic features are shown in Figure 1. Overall, more than 95.9% (6228 genes) of the protein-coding genes were annotated with at least one of the seven databases. In detail, a total of 2522 (38.9%), 3911 (60.3%), 4556 (70.3%), 4675 (72%), 4712 (72.6%), and 5959 (91.8%) protein-coding genes were matched with the COG, KOG, GO, Swiss-Prot, Pfam, and KEGG databases, respectively. Additionally, the annotated sequences were compared with the 6221 genes in the non-redundant (Nr) protein database, and the best-match results of the NR homologous species distribution are shown in Figure 3. The sequences of YZU573 showed the best match with Golovinomyces cichoracearum (653 genes), followed by Blumeria graminis (523 genes) and Erysiphe pulchra (363 genes). All three fungal species are the causal agents of plant PM disease [40]. These indicated the genes identified from the combination of the Illumina and nanopore sequencing provided comprehensive and accurate information at the genomic level for further clarifying the effector repertoire of *P. xanthii*.

### 3.4. Prediction of Effector Proteins

Of the 6491 protein-coding genes in the YZU573 genome, a total of 338 proteins were predicted to contain N-terminal signal peptides (SignalP v4.1). The 338 proteins were further scanned with TMHMM 2.0, and 100 proteins that contained transmembrane domains were removed from the protein data set. Of the remaining 238 proteins, 87 were identified as candidate effector proteins by EffectorP-fungi v3.0, including 54 cytoplasmic effectors, 16 apoplastic effectors, and 17 either as cytoplasmic or apoplastic effectors. The 87 candidate effector proteins were annotated by comparing them against COG/GO/KEGG/KOG/Pfam/Swiss-Prot/Nr databases, and 65 were annotated in at least one database. The remaining 22 effector proteins were novel and will need to be further evaluated for their roles in pathogenesis.

**Figure 4:**
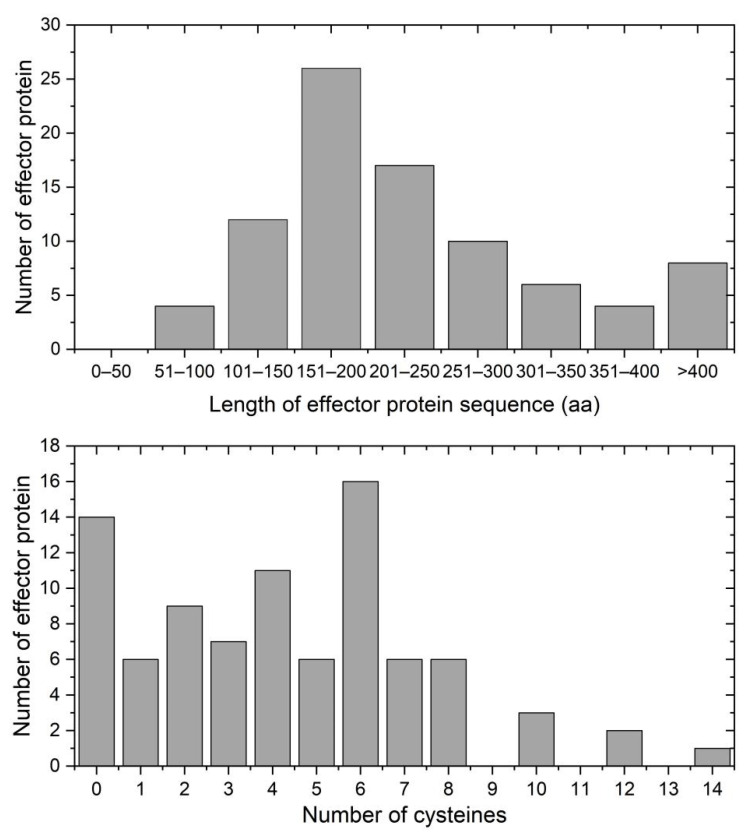
The features of *P. xanthii* effector proteins. Upper: lengths of the 87 effector proteins in the YZU573 genome. Lower: distribution of cysteines across the 87 candidate effector proteins identified in the YZU573 genome.

Analysis of amino acid sequences revealed that 79.3% (69 sequences) of *P. xanthii*. effector proteins were less than 300 aa in length (Figure 4), which is a common feature of effector proteins [41]. In addition, statistical analysis showed that 84 (96.5%) of the putative effector proteins contained 10 or fewer cysteines. Notably, 14 putative effector proteins lacked cysteines entirely, which included 11 cytoplasmic effectors, 2 apoplastic effectors, and 1 apoplastic/cytoplasmic effector (Figure 4). Cysteine content is typically applied to identify candidate apoplastic effectors, as high number of cysteines present in fungi likely form the disulfide bonds that appear to enhance effector stability in the apoplastic environment [42]. However, Huang et al. [43] found an apoplastic effector protein Fs05897 in *Fusarium sacchari* lacking cysteines entirely also showed the ability to induce plant cell death. Thus, the number of cysteines cannot be used as the sole criterion by which to identify effectors from fungal secretomes. 

## 4. Conclusions

We presented the genome sequences of the *P. xanthii* race 2F genome, isolated from cucumber leaves from China. The availability of this high-quality *P. xanthii* genome sequence data provide a fundamental resource to prioritize candidate effectors of interest for future studies. More important, these effectors can be used as tools to search for cucumber defense against *P. xanthii*, aiming to achieve new genetic resources with durable PM resistance.

## Figures and Tables

**Table 1:** Reactions of 13 melon accessions upon YZU573 inoculation in 3 years. Values are means of three replicates ± SD. DI: disease index. R: resistant. S: susceptible.

Accession	DI_2020_	DI_2021_	DI_2022_	Reaction Level
Iran H	75.6 ± 5.3	78.9 ± 1.9	53.6 ± 3.6	S
Top Mark	68.2 ± 2.0	86.7 ± 4.6	64.5 ± 3.7	S
PMR 5	2.2 ± 0.5	2.2 ± 1.1	3.2 ± 1.5	R
Vedrantais	82.5 ± 3.5	52.5 ± 4.0	80.8 ± 4.4	S
PI414723	0	0	0	R
PMR 45	112.3 ± 3.5	93.5 ± 5.6	107.7 ± 6.4	S
WMR29	0	0	0	R
MR-1	1.6 ± 1.1	0.5 ± 0.4	7.2 ± 1.1	R
PI124111	3.3 ± 1.3	4.1 ± 2.5	4.2 ± 1.8	R
Edisto47	0	0	0	R
PI124112	5.3 ± 1.0	2.4 ± 0.3	3.2 ± 1.1	R
PMR6	8.6 ± 1.6	2.1 ± 0.5	7.2 ± 1.6	R
Nantais Oblong	68.5 ± 3.5	63.5 ± 5.9	97.7 ± 5.4	S

**Table 2:** Genome assembly statistics for *P. xanthii* isolate YZU573.

Metric	Value
Assembly size (bp)	152,748,770
Number of contigs	58
Number of contigs (≥25,000 bp)	57
Number of contigs (≥50,000 bp)	48
Largest contig (bp)	14,306,468
GC (%)	43.27
N_50_ (bp)	6,916,385
L_50_	8
Number of Ns per 100 kb	0
BUSCOs (%)	
Complete BUSCOs (C)	99.2%
Complete and single-copy (S)	99.1%
Complete and duplicated (D)	0.1%
Fragmented (F)	0.4%
Missing (M)	0.4%
Gene	
protein-coding genes	6491
Average gene length (bp)	1563.8
Non-coding RNA	
tRNA	423
rRNA	12
sRNA	64

## Data Availability

The sequencing reads of *P. xanthii* isolate YZU573 that are described in this paper have been deposited at GenBank with the accession number PRJNA913294. The YZU573 strain is available upon request from the corresponding author. All the final products of analysis have been submitted to figshare (https://doi.org/10.6084/m9.figshare.21746645.v2, accessed on 10 March 2023) for public use.
